# Supplementary material for: Associations between psychosocial work environment factors and first-time and recurrent treatment for depression: a prospective cohort study of 24,226 employees
Source: Epidemiol Psychiatr Sci. 2024 Mar 18;33:e13. doi: 10.1017/S2045796024000167 (PMC10951797; doi:10.1017/S2045796024000167)
Supplement: Mathisen et al. supplementary material 1 — Mathisen et al. supplementary material [file S2045796024000167sup001.docx]

**ONLY FOR DOCUMENTING CHANGES – NOT TO BE PUBLISHED**

**ABSTRACT**

Aims
Adverse factors in the psychosocial work environment are associated with the onset of depression among those without a personal history of depression. However, the evidence is sparse regarding whether adverse work factors can also play a role in depression recurrence. This study aimed to prospectively examine whether factors in the psychosocial work environment are associated with first-time and recurrent treatment for depression.

Methods
The study included 24,226 participants from the Danish Well-being in Hospital Employees (WHALE) study. We measured ten individual psychosocial work factors and three theoretical constructs (effort-reward imbalance, job strain and workplace social capital). We ascertained treatment for depression through registrations of hospital contacts for depression (ICD-10: F32 and F33) and redeemed prescriptions of antidepressant medication (ATC: N06A) in Danish national registries. We estimated the associations between work factors and treatment for depression for up to two years after baseline among those without (first-time treatment) and with (recurrent treatment) a personal history of treatment for depression before baseline. We excluded participants registered with treatment within six months before baseline. In supplementary analyses, we extended this washout period to up to 2 years. We applied logistic regression analyses with adjustment for confounding.

Results
Among 21,156 (87%) participants without a history of treatment for depression, 350 (1.7%) had first-time treatment during follow-up. Among the 3,070 (13%) participants with treatment history, 353 (11%) had recurrent treatment during follow-up. Those with a history of depression generally reported a more adverse work environment than those without such a history. Baseline exposure to bullying (OR = 1.72, 95% CI: 1.30 – 2.32), and to some extent also low influence on work schedule (OR = 1.27, 95% CI: 0.97 – 1.66) and job strain (OR = 1.24, 95% CI: 0.97 – 1.57), was associated with first-time treatment for depression during follow-up. Baseline exposure to bullying (OR = 1.40, 95% CI: 1.04 – 1.88), lack of collaboration (OR = 1.31, 95% CI: 1.03 – 1.67) and low job control (OR = 1.27, 95% CI: 1.00 – 1.62) was associated with recurrent treatment for depression during follow-up. However, most work factors were not associated with treatment for depression. Using a two-year washout period resulted in similar or stronger associations.

Conclusions
Depression constitutes a substantial morbidity burden in the working-age population. Specific adverse working conditions were associated with first-time and recurrent treatment for depression, and improving these may contribute to reducing the onset and recurrence of depression.

**KEYWORDS:** Depression; Epidemiology; Prospective Study; Occupational Psychiatry

**INTRODUCTION**

Estimates suggest that 15% of working-age adults globally live with a mental disorder at any given time (WHO, 2022). Among these disorders, depression is the leading cause of disability worldwide (Vos *et al.*, 2020). In Europe and the USA, depression is the second-most common disorder (after anxiety disorders), with a 12-month prevalence of approximately 7% (Wittchen *et al.*, 2011; Kessler *et al.*, 2012). Around 50% of those developing a depressive episode will have at least one more episode in the future (or never remit) (Mattisson *et al.*, 2007; Eaton *et al.*, 2008).

Risk factors for the onset of depression include biological, psychological and social factors (Malhi & Mann, 2018) that may also include psychosocial factors in the work environment (WHO, 2022). Systematic reviews and meta-analyses have reported that specific psychosocial work factors are associated with the onset of depression (Theorell *et al.*, 2015; Madsen *et al.*, 2017; Rugulies *et al.*, 2017; Rudkjoebing *et al.*, 2020;Mikkelsen *et al.*, 2021;). These include, for example, job strain (Madsen *et al.*, 2017; Mikkelsen *et al.*, 20212021), effort-reward imbalance (Rugulies *et al.*, 2017; Mikkelsen *et al.*, 2021), low job control (Theorell *et al.*, 2015), workplace bullying (Theorell *et al.*, 2015; Mikkelsen *et al.*, 2021) and threats and violence at work (Rudkjoebing *et al.*, 2020).

To our knowledge, only three small-scale studies have reported prospective associations between psychosocial work factors and recurrent episodes of depression. First, a study of 583 Canadian working adults with a history of depression found that neither job strain, effort-reward imbalance, supervisor support, nor co-worker support was associated with the recurrence of depressive symptoms within a one-year follow-up period (Wang *et al.*, 2012). Second, a Japanese study of 540 employees returning to work after a leave of absence spell due to depression found that high job demands but not low job control were associated with a recurrent physician-certified episode of sickness absence due to depression (Endo *et al.*, 2015). Third, a US-American study included 233 participants who were employed at baseline and were under treatment for depression and followed their trajectory of depression of up to 23 years. Those with the least severe life course trajectory of depression had a higher work resource index at baseline (job involvement, social cohesion and supervisor support) (Heinz *et al.*, 2018). Consequently, there is a paucity of large-scale studies investigating whether work factors are associated with recurrent episodes of depression*.*

Major life stressors, such as loss of primary relationships or onset of serious illness, have a more substantial impact on the risk of first-time depressive episodes than subsequent episodes (Kessing & Andersen, 2017; Monroe *et al.*, 2019). Nevertheless, interpersonal stress and lack of social support (outside of work), and other stressors in everyday life, including at work (so-called daily hassles), have been associated with the recurrence of depression in most but not all studies (Bockting *et al.*, 2006; Buckman *et al.*, 2018) . These findings are in accordance with the stress sensitisation theory, the dominant theory of stress in recurrent depression, which posits that non-major stressors can trigger subsequent episodes ( . As such, it is plausible that stressors experienced at work could contribute to depression recurrence.

The potential link between psychosocial work factors and the recurrence of depression warrants further attention. Findings of associations between work factors and the recurrence of depression could inform guidelines regarding the prevention of depressive episodes in the workplace and thereby contribute to decreasing the enormous personal and societal costs of (recurrent) depression ( Chisholm *et al.*,; Christensen *et al.*, 2020; Malhi & Mann, 2018)).

Healthcare workers have been reported to face relatively adverse working conditions across several dimensions including, for example, high time pressure, high emotional work demands, and more exposure to violence and threats (Aagestad *et al.*, 2016; Vinckx *et al.*, 2018; ). In this paper, we used data on several dimensions of the psychosocial work environment in a large Danish public healthcare employee cohort linked to national register data on treatment for depression. We aimed to examine whether work factors are associated with the risk of first-time and recurrent treatment for depression.

**METHODS**

Study population

The study population consisted of participants in the 2014 wave of the Well-being in Hospital Employees (WHALE) study of all employees in the public healthcare enterprise of the Capital Region of Denmark in March 2014 (Hvidtfeldt *et al.*, 2017). Of the 37,720 participants invited to a workplace assessment survey, 31,823 (84%) responded. We excluded participants who: had inconsistent data (n=621); were trainees (n=107); worked less than 18.5 hours per week at baseline or were working on an hourly basis (n=612); had missing sociodemographic information (n=474); emigrated (n=230) or died (n=128) during follow-up or had missing values on any of the included psychosocial work factors (n=4086). We also excluded 1339 participants who had been treated for depression within the last six months before baseline to omit participants who concurrently or recently had undergone treatment for depression.

Participants treated for depression were identified through registrations of Anatomical Therapeutic Chemical (ATC) codes indicating redeeming of prescribed antidepressant medication (N06A) in the Danish National Prescription Registry (Pottegård *et al.*, 2016) and through registrations of the International Statistical Classification of Diseases and Related Health Problems version 10 (ICD-10) codes indicating a hospital contact with unipolar depression (F32 and F33) in the Danish National Patient Register (Lynge *et al.*, 2011). This definition thus includes participants with a depression-related contact with the public hospital sector, as well as participants treated using antidepressants in the primary and secondary care sector. Data on other forms of depression treatment was not available.

We defined two study populations based on the participants’ personal treatment histories. We used all the treatment data available in the project, dating back to 1 January 2000 (14.3 years), as recurrence of depression can occur up to 15 years or more after the recovery from an index episode (Mattisson *et al.*, 2007; Eaton *et al.*, 2008). Thus, we defined: I) A population of 21,156 participants without a registered history of treatment for depression within the past 14.3 years before baseline (1 January 2000 – 31 March 2014); II) A population of 3070 participants with a registered history of treatment for depression within the past 14.3 years, excluding those registered with treatment in the six-month washout period before baseline as described above (1 January 2000 – 31 August 2013). In total, the two study populations included 24,226 participants.

Psychosocial work factors

We examined ten individual work factors which we grouped into four broad dimensions: 1) Collegial relations included lack of collaboration and exposure to bullying (78% of those bullied reported the perpetrator to be a colleague or internal collaborator). 2) Job organisation included low job control, low influence over work schedule and high work demands. 3) Management and leadership concerning both the immediate supervisor (low leadership quality and low recognition) and the work unit in general (low justice and low trust). 4) Offensive behaviours by external actors included exposure to threats/violence (98% of those exposed to threats/violence reported the perpetrator to be a patient, user, relative or external collaborator, that is, persons external to the organisation). We also included three composite factors stemming from internationally recognised theoretical models within research on psychosocial work environments and health: Effort-reward imbalance (high work demands in combination with low recognition and justice) (Siegrist, 1996), job strain (a combination of high work demands and low job control) (Karasek, 1979), and low workplace social capital (a low average level of collaboration, justice and trust) (Kawachi & Berkman, 2014). Given the high number of included work factors, we dichotomised all factors to simplify their interpretation. Details of the measured work factors, including item phrasing, scale construction, construction of the composite measures, and exposure definitions are available in Appendix 1.

Treatment for depression

We followed the participants prospectively for registrations of treatment for depression for up to two years after baseline (1 April 2014 – 31 March 2016). We limited the follow-up period to two years to increase the likelihood that incident treatment for depression could be caused by the work exposures reported at baseline as changes in working conditions due to, for example, organisational changes are frequent within the Danish public hospital sector (Jensen *et al.*, 2018). We defined first-time treatment for depression as registrations of treatment occurring among those without a history of treatment and recurrent treatment for depression as registrations occurring among those with a history of treatment.

Covariates

We included sociodemographic and employment characteristics as covariates. The sociodemographic characteristics included age, sex, education, occupation, household income and marital status. Employment characteristics included seniority, full-time/part-time employment status and workplace (using the top-tier organisational structure in the Capital Region of Denmark). An overview of the categorisations is presented in Table 1. We obtained information regarding education, household income, and marital status from national registries, while we obtained information on age, sex and occupation from employer-based administrative data. We obtained all information on covariates at or before baseline (31 March 2013). Furthermore, we also included the number of years since the last treatment registration for those with a treatment history.

Statistical analysis

We performed the primary analyses separately in the two study populations. We applied logistic regression models to determine the associations between each psychosocial work factor and treatment for depression during follow-up. We applied Firth correction to correct a small degree of statistical separation in the logistic regression models. Furthermore, we weighted the regression models with inverse probability (IP) weights as we excluded a large number of participants due to missingness on at least one work factor (n=4086). We constructed these weights separately in the two study populations and modelled them as the inverse of the probability of having a missing value on at least one work factor conditional on the sociodemographic factors and employment characteristics. Thus, the IP weighting allowed the study populations to represent both those with and without missing work factor data. We report odds ratios (OR) with accompanying 95% confidence intervals (95% CI) from the logistic regression models. We adjusted all analyses for sociodemographic and employment characteristics, as mentioned above. Furthermore, we adjusted the analyses of recurrent treatment for depression for the time (years) passed since the last registered treatment.

We conducted three supplementary analyses: I) Single-factor analyses that were not IP-weighted. II) Mutually adjusted analyses. Mutually adjusting all work factors for each other will likely result in overadjustment due to their complex interplay, but they are reported here for completeness. III) The washout period of 6 months may not have adequately excluded participants treated with antidepressants at baseline. Therefore, we extended the washout period to 1 and 2 years, respectively.

**RESULTS**

In total, the study population included 24,226 participants, of which approximately four out of five were women. Overall, 3070 (13%) had a history of depression treatment, whereas 21,156 (87%) had not. Among those with a history, 92% were identified through redeemed prescriptions only (Supplementary Table 1). Table 1 shows the descriptive characteristics of the study populations.

Those with a history of treatment for depression were more often women and unmarried and had lower education and household income than those without a history. Also, a history of treatment was more frequent among social- and healthcare employees, pedagogical employees and administrative employees compared to other occupational groups.

In total, 703 participants (2.9%) registered with treatment for depression during the follow-up period. Of those, 95% were identified through redeemed prescriptions only (Supplementary Table 2). Among those at risk for first-time treatment, 350 (1.7%) had treatment for depression during follow-up, while among those at risk for recurrent treatment, 353 (11%) had treatment during follow-up. The crude risk of recurrent treatment was lower with more years without treatment (Supplementary Table 3).

Figure 1 shows the distributions of work factors among those with and without a treatment history at baseline. In general, participants with a treatment history reported more adverse psychosocial work environments on all factors than those without a history, most notably a higher exposure to bullying.

Figure 2 shows the associations between work factors and registering with first-time or recurrent treatment for depression during follow-up. Details of the estimates can be found in Supplementary Tables 4 and 5. The reference group of all associations are those not exposed to the specific risk factor. The odds of first-time treatment were higher only in those reporting exposure to bullying (OR = 1.72, 95% CI: 1.30 – 2.29). However, there was also a tendency towards higher odds of first-time treatment among participants who had a low influence on their work schedule (OR = 1.27, 95% CI: 0.97 – 1.66) and among those exposed to job strain (OR = 1.24, 95% CI: 0.97 – 1.57). The odds of recurrent treatment were higher among those experiencing bullying (OR = 1.40, 95% CI: 1.04 – 1.88) and a lack of collaboration (OR = 1.31, 95% CI: 1.03 – 1.67) and among those who had low job control (OR = 1.27, 95% CI: 1.00 – 1.63). Most work factors, however, were neither associated with first-time nor recurrent treatment for depression.

The absolute risk of depression treatment during follow-up was much higher for those at risk of recurrent treatment (11%) than those at risk of first-time treatment (1.7%). Accordingly, among those under risk for first-time treatment who were bullied, the absolute risk was 2.8%, while among those under risk for recurrent treatment who were bullied, the absolute risk was 15%. Due to the substantial differences in absolute risk, the odds ratios from the two analyses are not directly comparable.

Supplementary analyses

The estimates in the analyses without IP-weighting were similar to the primary analysis (Supplementary Tables 6 and 7). The estimates in the mutually adjusted analyses were also similar to the primary analyses. However, all confidence intervals overlapped unity in the analyses of recurrence treatment (Supplementary Tables 8 and 9). In analyses of recurrent treatment, where the exclusion of participants treated before baseline was extended to 1 and 2 years, respectively, the associations for exposure to bullying (OR_2Y_ = 1.85, 95% CI: 1.30 – 2.63) and low job control (OR_2Y_ = 1.37, 95% CI: 1.02 – 1.85) became stronger with longer exclusion time. Most other point estimates were similar to the primary analysis. However, the confidence intervals became wider due to fewer cases (Supplementary Tables 10 and 11).

**DISCUSSION**

Thirteen percent of the employees had a history of treatment for depression. Among them, more than one in ten had recurrent treatment within the 2-year follow-up period. In general, those with a history of treatment perceived their psychosocial work environment more negatively than those without depression. Overall, we found that only few work factors were associated with treatment for depression.

Exposure to bullying and, to some extent, job strain were associated with a higher risk of first-time treatment for depression. In previous studies, bullying was generally the strongest psychosocial work risk factor for the onset of depression, with a more than 2-fold higher risk previously reported (Theorell *et al.*, 2015; Mikkelsen *et al.*, 2021). The association between job strain and first-time treatment for depression reported here is similar in magnitude to those reported in some analyses (Madsen *et al.*, 2017; Mikkelsen *et al.*, 2021) and lower than in other analyses (Madsen *et al.*, 2017). The confidence intervals reported here likely overlap unity due to a low number of cases. Thus, the findings regarding bullying job strain are generally in line with previous studies.

We did not find an association between low job control and first-time treatment for depression, even though this association has previously been reported (Theorell *et al.*, 2015; Madsen *et al.*, 2017; Mikkelsen *et al.*, 2021). The point estimate reported in this study is similar to the one reported in the systematic review by Mikkelsen et al., but had wider confidence intervals (Mikkelsen *et al.*, 2021). The association reported by Madsen et al. was based on more cases and longer follow-up (Madsen *et al.*, 2017) while the summary estimate from the meta-analysis by Theorell et al. was based on the least adjusted models in the primary studies, which might not have been adequately controlled for confounding (Theorell *et al.*, 2015). Thus, these discrepancies may be due to various methodological differences between this study and the previous meta-analyses. Also contrary to prior literature, we did not find an association between exposure to violence and threats and first-time treatment for depression (Rudkjoebing *et al.*, 2020), nor between effort-reward imbalance and first-time treatment for depression (Rugulies *et al.*, 2017; Mikkelsen *et al.*, 2021).

Regarding recurrent treatment, we found that exposure to bullying, a lack of collaboration and low job control were associated with recurrent treatment for depression. In line with our findings, a more supportive work environment at baseline was associated with a less severe life-course trajectory of depression in a US-American study of 233 working adults with depression (Heinz *et al.*, 2018). However, low co-worker support was not associated with depressive symptoms after one year in a small Canadian cohort study of 583 working adults with a history of depression (Wang *et al.*, 2012). Endo et al. reported that high job demands but not low job control were associated with recurrent physician-certified episodes of sickness absence due to depression among 540 Japanese employees with previous registrations of such a sickness absence episode (Endo *et al.*, 2015). In contrast, we found that low job control, but not high job demands were associated with recurrent treatment for depression. None of the previous studies included exposure to bullying, which showed the strongest association with both first-time and recurrent treatment in the present study.

**Interpretation and implications**

The stress sensitisation theory posits that non-major stressors, which might not be strong enough to trigger an initial episode of depression, may trigger recurrent episodes of depression as individuals become sensitised to stress after a first-time depressive episode ( Monroe *et al.*, 2019). It seems reasonable to assume that workplace bullying can be considered a major stressor (Mikkelsen *et al.*, 2020). Accordingly, we found that bullying was associated with both first-time and recurrent treatment for depression. However, although results from selected analyses (on lack of collaboration and low job control) agree with the stress sensitisation theory, overall, our study does not provide strong support for this theory within a workplace setting as most work stressors were not associated with recurrent treatment for depression. Nevertheless, these findings align with studies suggesting that interpersonal stressors, lack of social support outside work, and daily life stressors (including those at work) can affect the risk of recurrent episodes of depression (Bockting *et al.*, 2006; Buckman *et al.*, 2018).

This study was conducted among predominantly female public hospital employees. Women have higher incidence of depression than men (Wittchen *et al.*, 2011; Kessler *et al.*, 2012), but there is only limited evidence for differential associations between risk factors and depression between women and men (Kuehner, 2017). Healthcare employees may be less prone to seek treatment for mental health problems, due to, for example, concerns over confidentiality or negative social judgment (Clement *et al.*, 2015). This suggests that the associations between work factors and treatment for depression reported here might be slightly underestimated compared to other occupations or to, for example, a nationally representative sample of employees.

**Strengths and limitations**

Strengths of the study include the large study population with a very high response rate, comprehensive measurement of several psychosocial work factors and prospective linkage to national registers. Employing different data sources, we could assess the associations between several work factors and both first-time and recurrent treatment for depression while adjusting for sociodemographic and employment factors. The study adds much-needed evidence regarding the role of work factors in recurrent depression.

Several limitations are relevant to discuss. First, register-based treatment information is not optimal for identifying those who develop depressive episodes. Fewer than half of those with a depressive episode are in contact with the healthcare system (Packness *et al.*, 2018), and even fewer are treated with antidepressant medication (Packness *et al.*, 2018). A recent Danish study found that among those who screened positive for depression using the Major Depression Inventory, 51% had redeemed a prescription of antidepressant medication within ten years before to two years after the screening (Weye *et al.*, 2023). In this study, most cases were registered solely by the criteria of having redeemed prescribed antidepressant medication. Consequently, our estimates of the incidence and prevalence of treatment for depression are likely underestimated compared to the actual rates of depression. Furthermore, antidepressant medication is commonly taken for other mental disorders, such as anxiety/panic disorders or sleep disorders (Kazdin *et al.*, 2023). Thus, we have likely misclassified some participants who are being treated for other disorders as being treated for depression.

Second, we were not able to account for the nature of the participants’ history of depression at baseline, which is among the strongest predictors of the recurrence. This includes whether residual symptoms are present (Buckman *et al.*, 2018), the severity of the first episode (Buckman *et al.*, 2018) and the number of previous episodes (Kessing & Andersen, 2017; Buckman *et al.*, 2018). Concurrent depression or depressive symptoms at baseline may have negatively influenced the appraisal of the work environment and increased the risk of being treated for depression during follow-up. Thereby, this source of confounding could have biased the results away from the null. However, the associations with recurrent treatment were similar (or stronger) when we extended the exclusion period before baseline for up to two years, excluding those with the highest risk of recurrence.

Third, the study population with a depression treatment history are likely a selected sample of all working-age individuals with a depression history. Mental health issues such as depression are associated with involuntary exit from the workplace to unemployment or disability pension (Rijn *et al.*, 2014). Furthermore, those with more severe episodes of depression are perhaps less likely to enter, stay at or return to work. Similarly, those with more adverse work environments are perhaps also less likely to stay or return than those with more favourable work environments. Such selection forces likely biased the associations for recurrent treatment towards the null.

Finally, some of the work factors were measured using a limited number of items. In particular, the measure of effort-reward imbalance may have been measured inadequately as we were only able to include items covering recognition from the management and the experience of justice as rewards while monetary rewards or job security also form part of the original effort-reward imbalance construct (Siegrist, 1996). A similar point can be raised about other work factors, for example, *justice* (often referred to as procedural justice), which originally was measured using seven items whereas we measured it using only two items (Elovainio *et al.*, 2002). However, one multi-cohort study has suggested that using one-item measurements of procedural justice may not differ much from using instruments with more items (Xu *et al.*, 2022).

**Conclusions**

Depression constitutes a substantial morbidity burden in the workplace. Thirteen percent of the participants had a history of treatment for depression, and they experienced more adverse work environments and had a much higher risk of treatment for depression during follow-up compared to those without such a history. Exposure to bullying was associated with higher odds of both first-time and recurrent treatment for depression. Furthermore, job strain and low influence over the work schedule were associated with first-time treatment for depression, while low job control and lack of collaboration were associated with recurrent treatment for depression.

**REQUIRED STATEMENTS**

**Acknowledgements**

None

**Financial support**

The work was supported by a grant from the Danish Regions (employer organisation), The Danish Association of Local Government Employees Organizations (Forhandlingsfællesskabet; employee organisation) and the Danish Working Environment Research Fund (grant number 14-2018-09). The sponsors had no role in the study design, the collection, analysis and interpretation of the data, the writing of the manuscript or the decision to submit it for publication.

**Conflict of interest**JM is currently employed at Novo Nordisk A/S and affiliated as a guest researcher at the Section of Epidemiology at the University of Copenhagen. The majority of the work by JM on this manuscript was conducted during a former employment spell at the Section of Epidemiology at the University of Copenhagen, and the remaining work was conducted in the role as a guest researcher at the University of Copenhagen. Novo Nordisk A/S had no role in the study design, the collection, analysis and interpretation of the data, the writing of the manuscript or the decision to submit it for publication. The other authors report no conflict of interest.

**Ethical Standards**

The authors assert that all procedures contributing to this work comply with the ethical standards of the relevant national and institutional committees on human experimentation and with the Helsinki Declaration of 1975, as revised in 2000.

**Data availability**Data used in this study and from Danish registers are not publicly available due to Danish legislation. Anonymised data from the WHALE study are available upon reasonable request through collaborative agreements. Please contact Professor Naja Hulvej Rod [nahuro@sund.ku.dk] for further details.

**FIGURE CAPTIONS**

Figure 1. Distribution of work factors among those with (N = 3070) and without (N= 21,156) history of treatment for depression at baseline.

Figure 2. Associations between psychosocial work factors and first-time (N = 21,156) and recurrent treatment for depression (N = 3070). Numerical figures are shown in Supplementary Tables 4 and 5.

**Figure 2 legend (to be printed below the figure):**The analyses for first-time treatment were adjusted for sex, age, marital status, education, household income, occupation, workplace, seniority, full-time/part-time status. The analyses for recurrent treatment were adjusted for the same as the analyses for first-time treatment + time since the last treatment.
