# Supplementary material for: Associations between psychosocial work environment factors and first-time and recurrent treatment for depression: a prospective cohort study of 24,226 employees
Source: Epidemiol Psychiatr Sci. 2024 Mar 18;33:e13. doi: 10.1017/S2045796024000167 (PMC10951797; doi:10.1017/S2045796024000167)
Supplement: Mathisen et al. supplementary material 2 — Mathisen et al. supplementary material [file S2045796024000167sup002.docx]

**SUPPLEMENTARY MATERIALS**

**Title:** Associations between psychosocial work environment factors and first-time and recurrent treatment for depression: A prospective cohort study of 24,226 employees.

**Running title:** Psychosocial work factors and treatment for depression

**Authors:** J Mathisen^1,2^, T-L Nguyen^1^, IEH Madsen^2,3,4^, T Xu^5^, JH Jensen^2,6^, JK Sørensen^3^, R Rugulies^1,2,3^, NH Rod^1^

**Affiliations:**

1: Section of Epidemiology, Department of Public Health, University of Copenhagen, Copenhagen, Denmark

2: Copenhagen Stress Research Center, Copenhagen, Denmark

3: National Research Centre for the Working Environment, Copenhagen, Denmark

4: The National Institute of Public Health, University of Southern Denmark, Copenhagen, Denmark

5: Stress Research Institute, Department of Psychology, Stockholm University, Stockholm, Sweden

6: Department of Occupational and Environmental Medicine, Copenhagen University Hospital – Bispebjerg and Frederiksberg, Copenhagen, Denmark

**Contents:**

Appendix 1: Details of measured psychosocial work factors and composite measures.

Appendix 2: Supplementary tables

**Corresponding author:**

Name: Jimmi Mathisen
Institution: Section of Epidemiology, Department of Public Health, University of Copenhagen

Address: Oester Farimagsgade 5, P.O. Box 2099, 1014 Copenhagen, Denmark

Telephone number: +45 35 33 53 39

Email: [jima@sund.ku.dk](mailto:jima@sund.ku.dk)

ORCID: 0000-0002-7391-7296

**Appendix 1: Details of measured psychosocial work factors and composite measures**

| Interpersonal relations with colleagues | | |
| --- | --- | --- |
| Bullying |  | Have you been exposed to bullying within the last 12 months? If yes, how often? |
| Collaboration | How often… | … are you and your colleagues good at coming up with suggestions for improving work procedures? |
|  |  | … are you and your colleagues take responsibility for a nice atmosphere and tone of communication? |
|  |  | … do you get help and support from your colleagues when needed? |
| Job organization | | |
| Control | To what extent… | … do you have influence on how you do your work? |
|  |  | … do you have the opportunity of learning new things through your work? |
| Schedule influence | To what extent… | … are you able to schedule your work time, so you can take into account private matters? |
| Work demands | How often… | … do you have time for breaks during your work day? |
|  |  | … do you have enough time for your work tasks? |
| Management and leadership | | |
| Leadership quality | To what extent… | … is <nearest supervisor> good at work planning? |
|  |  | … does <nearest supervisor> give high priority to job satisfaction? |
|  |  | … do you get help and support from your nearest supervisor when needed? |
| Recognition | To what extent… | … is your work recognized and appreciated by your nearest supervisor? |
| Justice | To what extent… | … are conflicts resolved in a fair way? |
|  |  | … is the work distributed fairly? |
| Trust | How To what extent.. | … does the management trust the employees to do their work well? |
|  |  | … can you trust the information that comes from the management? |
| Offensive behaviors (external actors) | | |
| Violence and threats |  | Have you been exposed to violence within the last 12 months? If yes, how often? |
|  |  | Have you been exposed to threats within the last 12 months? If yes, how often? |

**Construction of composite measures**

Job Strain

Job strain is defined as a combination between high work demands and low decision latitude (control over work) Low decision latitude combines measures of influence on work and possibilities for development. (1)

Included items:

| Work demands. How often… |
| --- |
| …do you have time for breaks throughout your workday? |
| …do you have enough time for your work tasks? |
| Job control. How often… |
| …do you have influence on how you do your work? |
| …do you have the possibility of learning new things through your work? |

Variable construction:

We calculated the mean of work demands and mean of control. If employees have higher than median mean work demands and lower than median mean control, then employees are exposed to job strain.

Effort-reward imbalance

Effort-reward imbalance is defined as an imbalance between work demands and rewards in terms of money, job security and esteem (2,3). Here, we only have one item available which directly measures recognition. However, Siegrist et al. (2004) points out that rewards also encompass also defines a lack of rewards as: “*Feelings of not being appreciated in an adequate way or of being treated unfairly and disappointments resulting from inappropriate rewards”* (3). Therefore, also two items from the justice scale are also included as rewards.

Included items:

| Work demands. How often… |
| --- |
| … do have time for breaks throughout your workday? |
| … do you have enough time for your work tasks? |
| Rewards. How often… |
| … is your work recognized and appreciated by the management? |
| … are conflicts resolved in a fair way? |
| To what extent is the work distributed fairly? |

Variable construction:

We calculated the average score of work demand items and the average score on reward items. If the ratio of *effort* / *reward* is above 1.0, then employees are considered exposed to effort-reward imbalance.

**Workplace social capital**

Social capital was defined in accordance with previous use in the WHALE cohort, encompassing items measuring trust, justice and collaboration (4) The construct measure both horizontal (relations between employees at the same hierarchical level) and vertical components (relations between employees at different hierarchical levels).

Included items:

| Trust. To what extent… |
| --- |
| … does the management trust the employees to do their work well? |
| … can you trust the information that comes from the management? |
| Justice. To what extent… |
| … are conflicts resolved in a fair way? |
| … is the work distributed fairly? |
| Collaboration. To what extent… |
| … are you and your colleagues good at coming up with suggestions for improving work procedures? |
| … are you and your colleagues take responsibility for a nice atmosphere and tone of communication? |
| … do you get help and support from your colleagues when needed? |

Variable construction:

In accordance with previous use, we calculated the average score across all eight items (4). Employees were defined as exposed to low social capital, if their average score fell at or below the 25% percentile across the sample.

**REFERENCES**

1. Fransson EI. Comparison of alternative versions of the job demand-control scales in 17 European cohort studies: the IPD-Work consortium. 2012;9.

2. Siegrist J, Dragano N, Nyberg ST, Lunau T, Alfredsson L, Erbel R, et al. Validating abbreviated measures of effort-reward imbalance at work in European cohort studies: the IPD-Work consortium. Int Arch Occup Environ Health. 2014;8.

3. Siegrist J, Starke D, Chandola T, Godin I, Marmot M, Niedhammer I, et al. The measurement of effort–reward imbalance at work: European comparisons. Social Science. 2004;17.

4. Hvidtfeldt UA, Bjorner JB, Jensen JH, Breinegaard N, Hasle P, Bonde JPE, et al. Cohort Profile: The Well-being in HospitAL Employees (WHALE) study. Int J Epidemiol. 2017 Dec 1;46(6):1758–1759h.

**Appendix 2: Supplementary tables**

|  | **Antidepressants** | | |
| --- | --- | --- | --- |
| **Hospital treatment** | No | Yes | Total |
| No | 0  (0.0%) | 2817 (91.8%) | 2817 (91.8%) |
| Yes | 41  (1.3%) | 212 (6.9%) | 253  (8.2%) |
| Total | 41  (1.3%) | 3029 (98.7%) | 3070 (100.0%) |

**Supplementary Table 1.** Distributions of types of treatment for depression registered before baseline for subjects with a history of depression (N = 3070).

**Supplementary Table 2.** Distributions of types of treatment for depression registered in the follow-up period (N = 703).

|  | **Antidepressants** | | |
| --- | --- | --- | --- |
| **Hospital treatment** | No | Yes | Total |
| No | 0  (0.0%) | 669 (95.2%) | 669 (95.2%) |
| Yes | 5  (0.7%) | 29 (4.1%) | 34  (4.8%) |
| Total | 5  (0.7%) | 698 (99.3%) | 703 (100.0%) |

**Supplementary Table 3.** Crude absolute risk of treatment for depression during follow-up according to number of treatment-free years before baseline.

|  | N | N treatment during follow-up | Crude Risk, % | 95% CI  lower | 95% CI  upper |
| --- | --- | --- | --- | --- | --- |
| **No history, total** | 21156 | 350 | 1.7 | 1.5 | 1.8 |
| **With history, total** | 3070 | 353 | 11.5 | 10.4 | 12.6 |
| **Number of treatment-free years among those with history** |  |  |  |  |  |
| 0.5 – 0.99 | 150 | 56 | 37.3 | 29.6 | 45.1 |
| 1 – 1.99 | 365 | 91 | 24.9 | 20.5 | 29.4 |
| 2 – 2.99 | 310 | 37 | 11.9 | 8.3 | 15.5 |
| 3 – 3.99 | 319 | 32 | 10.0 | 6.7 | 13.3 |
| 4 – 4.99 | 276 | 28 | 10.1 | 6.6 | 13.7 |
| 5 – 5.99 | 224 | 20 | 89.3 | 5.2 | 12.7 |
| 6 – 6.99 | 231 | 23 | 10.0 | 6.1 | 13.8 |
| 7 – 7.99 | 234 | 15 | 6.4 | 3.3 | 9.6 |
| 8 – 8.99 | 207 | 15 | 7.2 | 1.8 | 10.8 |
| 9 – 9.99 | 177 | 14 | 7.9 | 3.9 | 11.9 |
| 10 – 10.99 | 187 | 7 | 3.7 | 1.0 | 6.5 |
| 11 or more^a^ | 390 | 15 | 3.8 | 1.9 | 5.8 |

a Participants with 11 or more treatment-free years was grouped because of small cell numbers among those with more than 11 treatment-free years (<5 observations).

**Supplementary Table 4.** IP-weighted associations between psychosocial work factors and first-time treatment for depression (N = 21,156).

|  | Exposure status | n | % | Cases | Risk | Crude risk ratio | Adjusted odds ratio | 95% CI  lower | 95% CI  upper |
| --- | --- | --- | --- | --- | --- | --- | --- | --- | --- |
| Total | - | 21156 | 100 | 350 | 1,7 | - | - | - | - |
| Interpersonal relations |  |  |  |  |  |  |  |  |  |
|  | No | 19119 | 90 | 293 | 1,5 | Ref. | **-** | - | - |
| Bullying | Yes | 2037 | 10 | 57 | 2,8 | 1.83 | 1.72 | 1.30 | 2.29 |
|  | No | 15894 | 75 | 251 | 1,6 | Ref. | - | - | - |
| Lack of collaboration | Yes | 5262 | 25 | 99 | 1,9 | 1.19 | 1.12 | 0.89 | 1.42 |
| Job organisation |  |  |  |  |  |  |  |  |  |
|  | No | 16053 | 76 | 256 | 1,6 | Ref. | - | - | - |
| Low control | Yes | 5103 | 24 | 94 | 1,8 | 1.16 | 1.07 | 0.84 | 1.35 |
|  | No | 18019 | 85 | 284 | 1,6 | Ref. | - | - | - |
| Low influence on schedule | Yes | 3137 | 15 | 66 | 2,1 | 1.33 | 1.27 | 0.97 | 1.66 |
|  | No | 13454 | 64 | 212 | 1,6 | Ref. | - | - | - |
| High work demands | Yes | 7702 | 36 | 138 | 1,8 | 1.14 | 1.14 | 0.92 | 1.41 |
| Management and leadership |  |  |  |  |  |  |  |  |  |
|  | No | 16122 | 76 | 254 | 1,6 | Ref. | - | - | - |
| Low justice | Yes | 5034 | 24 | 96 | 1,9 | 1.21 | 1.14 | 0.90 | 1.45 |
|  | No | 15884 | 75 | 265 | 1,7 | Ref. | - | - | - |
| Low leadership quality | Yes | 5272 | 25 | 85 | 1,6 | 0.97 | 0.95 | 0.75 | 1.21 |
|  | No | 14229 | 67 | 247 | 1,7 | Ref. | - | - | - |
| Low recognition | Yes | 6927 | 33 | 103 | 1,5 | 0.86 | 0.84 | 0.67 | 1.05 |
|  | No | 16576 | 78 | 274 | 1,7 | Ref. | - | - | - |
| Low trust | Yes | 4580 | 22 | 76 | 1,7 | 1.00 | 0.97 | 0.75 | 1.25 |
| Offensive behaviour by external actors |  |  |  |  |  |  |  |  |  |
|  | No | 18038 | 85 | 287 | 1,6 | Ref. | - | - | - |
| Threats and violence | Yes | 3118 | 15 | 63 | 2,0 | 1.27 | 1.16 | 0.86 | 1.58 |
| Composite variables |  |  |  |  |  |  |  |  |  |
|  | No | 16790 | 79 | 285 | 1,7 | Ref. | - | - | - |
| Effort-reward imbalance | Yes | 4366 | 21 | 65 | 1,5 | 0.88 | 0.83 | 0.63 | 1.08 |
|  | No | 16665 | 79 | 260 | 1,6 | Ref. | - | - | - |
| Job strain | Yes | 4491 | 21 | 90 | 2,0 | 1.28 | 1.24 | 0.97 | 1.57 |
|  | No | 16310 | 77 | 269 | 1,6 | Ref. | - | - | - |
| Low social capital | Yes | 4846 | 23 | 81 | 1,7 | 1.01 | 0.96 | 0.75 | 1.23 |

All analyses were adjusted for age, sex, marital status, education, household income, occupation seniority and part/full-time status.
All analyses were weighted by the inverse probability of having missing values on at least one work-factor conditional on age, sex, marital status, education, household income, occupation, seniority, and part/full-time status.

**Supplementary Table 5.** IP-weighted associations between psychosocial work factors and recurrent treatment for depression (N = 3070).

|  | Exposure status | n | % | Cases | Risk | Crude risk ratio | Adjusted odds ratio | 95% CI  lower | 95% CI  upper |
| --- | --- | --- | --- | --- | --- | --- | --- | --- | --- |
| Total | - | 21156 | 100 | 350 | 1.7 | - | - | - | - |
| Interpersonal relations |  |  |  |  |  |  |  |  |  |
|  | No | 2623 | 85 | 284 | 10.8 | Ref | - | - | - |
| Bullying | Yes | 447 | 15 | 69 | 15.4 | 1.43 | 1.40 | 1.04 | 1.88 |
|  | No | 2174 | 71 | 231 | 10.6 | Ref | - | - | - |
| Lack of collaboration | Yes | 896 | 29 | 122 | 13.6 | 1.28 | 1.31 | 1.03 | 1.67 |
| Job organisation |  |  |  |  |  |  |  |  |  |
|  | No | 2161 | 70 | 230 | 10.6 | Ref | - | - | - |
| Low control | Yes | 909 | 30 | 123 | 13.5 | 1.27 | 1.27 | 1.00 | 1.62 |
|  | No | 2543 | 83 | 282 | 11.1 | Ref | - | - | - |
| Low influence on schedule | Yes | 527 | 17 | 71 | 13.5 | 1.21 | 1.19 | 0.89 | 1.59 |
|  | No | 1849 | 60 | 222 | 12.0 | Ref | - | - | - |
| High work demands | Yes | 1221 | 40 | 131 | 10.7 | 0.89 | 0.91 | 0.72 | 1.15 |
| Management and leadership |  |  |  |  |  |  |  |  |  |
|  | No | 2213 | 72 | 246 | 11.1 | Ref | - | - | - |
| Low justice | Yes | 857 | 28 | 107 | 12.5 | 1.12 | 1.15 | 0.89 | 1.47 |
|  | No | 2198 | 72 | 258 | 11.7 | Ref | - | - | - |
| Low leadership quality | Yes | 872 | 28 | 95 | 10.9 | 0.93 | 0.88 | 0.68 | 1.14 |
|  | No | 1915 | 62 | 217 | 11.3 | Ref | - | - | - |
| Low recognition | Yes | 1155 | 38 | 136 | 11.8 | 1.04 | 1.04 | 0.83 | 1.32 |
|  | No | 2268 | 74 | 258 | 11.4 | Ref | - | - | - |
| Low trust | Yes | 802 | 26 | 95 | 11.8 | 1.04 | 1.03 | 0.80 | 1.34 |
| Offensive behaviour by external actors |  |  |  |  |  |  |  |  |  |
|  | No | 2542 | 83 | 291 | 11.4 | Ref | - | - | - |
| Threats and violence | Yes | 528 | 17 | 62 | 11.7 | 1.03 | 1.04 | 0.74 | 1.45 |
| Composite variables |  |  |  |  |  |  |  |  |  |
|  | No | 2239 | 73 | 264 | 11.8 | Ref | - | - | - |
| Effort-reward imbalance | Yes | 731 | 24 | 89 | 12.2 | 1.03 | 1.01 | 0.78 | 1.32 |
|  | No | 2298 | 75 | 266 | 11.6 | Ref | - | - | - |
| Job strain | Yes | 772 | 25 | 87 | 11.3 | 0.97 | 0.99 | 0.76 | 1.29 |
|  | No | 2185 | 71 | 240 | 11.0 | Ref | - | - | - |
| Low social capital | Yes | 885 | 29 | 113 | 12.8 | 1.16 | 1.17 | 0.91 | 1.49 |

All analyses were adjusted for age, sex, marital status, education, household income, occupation, workplace, seniority, part/full-time status, and number of years since last treatment.
All analyses were weighted by the inverse probability of having missing values on at least one work-factor conditional on age, sex, marital status, education, household income, occupation, seniority and part/full-time status.

**Supplementary Table 6.** Unweighted associations between psychosocial work factors and first-time treatment for depression (N = 21,156).

|  | Exposure status | n | % | Cases | Risk | Crude risk ratio | Adjusted odds ratio | 95% CI  lower | 95% CI  upper |
| --- | --- | --- | --- | --- | --- | --- | --- | --- | --- |
| Total | - | 21156 | 100 | 350 | 1,7 | - | - | - | - |
| Interpersonal relations |  |  |  |  |  |  |  |  |  |
|  | No | 19119 | 90 | 293 | 1,5 | Ref. | **-** | - | - |
| Bullying | Yes | 2037 | 10 | 57 | 2,8 | 1.83 | 1.73 | 1.30 | 2.30 |
|  | No | 15894 | 75 | 251 | 1,6 | Ref. | **-** | - | - |
| Lack of collaboration | Yes | 5262 | 25 | 99 | 1,9 | 1.19 | 1.14 | 0.90 | 1.44 |
| Job organisation |  |  |  |  |  |  |  |  |  |
|  | No | 16053 | 76 | 256 | 1,6 | Ref. | **-** | - | - |
| Low control | Yes | 5103 | 24 | 94 | 1,8 | 1.16 | 1.08 | 0.85 | 1.37 |
|  | No | 18019 | 85 | 284 | 1,6 | Ref. | **-** | - | - |
| Low influence on schedule | Yes | 3137 | 15 | 66 | 2,1 | 1.33 | 1.27 | 0.97 | 1.66 |
|  | No | 13454 | 64 | 212 | 1,6 | Ref. | **-** | - | - |
| High work demands | Yes | 7702 | 36 | 138 | 1,8 | 1.14 | 1.14 | 0.92 | 1.41 |
| Management and leadership |  |  |  |  |  |  |  |  |  |
|  | No | 16122 | 76 | 254 | 1,6 | Ref. | **-** | - | - |
| Low justice | Yes | 5034 | 24 | 96 | 1,9 | 1.21 | 1.15 | 0.91 | 1.46 |
|  | No | 15884 | 75 | 265 | 1,7 | Ref. | **-** | - | - |
| Low leadership quality | Yes | 5272 | 25 | 85 | 1,6 | 0.97 | 0.95 | 0.74 | 1.20 |
|  | No | 14229 | 67 | 247 | 1,7 | Ref. | **-** | - | - |
| Low recognition | Yes | 6927 | 33 | 103 | 1,5 | 0.86 | 0.83 | 0.66 | 1.05 |
|  | No | 16576 | 78 | 274 | 1,7 | Ref. | **-** | - | - |
| Low trust | Yes | 4580 | 22 | 76 | 1,7 | 1.00 | 0.96 | 0.74 | 1.24 |
| Offensive behaviour by external actors |  |  |  |  |  |  |  |  |  |
|  | No | 18038 | 85 | 287 | 1,6 | Ref. | **-** | - | - |
| Threats and violence | Yes | 3118 | 15 | 63 | 2,0 | 1.27 | 1.16 | 0.85 | 1.57 |
| Composite variables |  |  |  |  |  |  |  |  |  |
|  | No | 16790 | 79 | 285 | 1,7 | Ref. | **-** | - | - |
| Effort-reward imbalance | Yes | 4366 | 21 | 65 | 1,5 | 0.88 | 0.84 | 0.64 | 1.09 |
|  | No | 16665 | 79 | 260 | 1,6 | Ref. | **-** | - | - |
| Job strain | Yes | 4491 | 21 | 90 | 2,0 | 1.28 | 1.24 | 0.98 | 1.58 |
|  | No | 16310 | 77 | 269 | 1,6 | Ref. | **-** | - | - |
| Low social capital | Yes | 4846 | 23 | 81 | 1,7 | 1.01 | 0.96 | 0.75 | 1.24 |

All analyses were adjusted for age, sex, marital status, education, household income, occupation seniority and part/full-time status, seniority, and part/full-time status.

**Supplementary Table 7.** Unweighted associations between psychosocial work factors and recurrent treatment for depression (N = 3070).

|  | Exposure status | n | % | Cases | Risk | Crude risk ratio | Adjusted odds ratio | 95% CI  lower | 95% CI  upper |
| --- | --- | --- | --- | --- | --- | --- | --- | --- | --- |
| Total | - | 21156 | 100 | 350 | 1.7 | - | - | - | - |
| Interpersonal relations |  |  |  |  |  |  |  |  |  |
|  | No | 2623 | 85 | 284 | 10.8 | Ref | - | - | - |
| Bullying | Yes | 447 | 15 | 69 | 15.4 | 1.43 | 1.41 | 1.05 | 1.89 |
|  | No | 2174 | 71 | 231 | 10.6 | Ref | - | - | - |
| Lack of collaboration | Yes | 896 | 29 | 122 | 13.6 | 1.28 | 1.30 | 1.02 | 1.65 |
| Job organisation |  |  |  |  |  |  |  |  |  |
|  | No | 2161 | 70 | 230 | 10.6 | Ref | - | - | - |
| Low control | Yes | 909 | 30 | 123 | 13.5 | 1.27 | 1.27 | 1.00 | 1.63 |
|  | No | 2543 | 83 | 282 | 11.1 | Ref | - | - | - |
| Low influence on schedule | Yes | 527 | 17 | 71 | 13.5 | 1.21 | 1.18 | 0.88 | 1.58 |
|  | No | 1849 | 60 | 222 | 12.0 | Ref | - | - | - |
| High work demands | Yes | 1221 | 40 | 131 | 10.7 | 0.89 | 0.90 | 0.71 | 1.14 |
| Management and leadership |  |  |  |  |  |  |  |  |  |
|  | No | 2213 | 72 | 246 | 11.1 | Ref | - | - | - |
| Low justice | Yes | 857 | 28 | 107 | 12.5 | 1.12 | 1.15 | 0.90 | 1.48 |
|  | No | 2198 | 72 | 258 | 11.7 | Ref | - | - | - |
| Low leadership quality | Yes | 872 | 28 | 95 | 10.9 | 0.93 | 0.89 | 0.69 | 1.15 |
|  | No | 1915 | 62 | 217 | 11.3 | Ref | - | - | - |
| Low recognition | Yes | 1155 | 38 | 136 | 11.8 | 1.04 | 1.05 | 0.83 | 1.32 |
|  | No | 2268 | 74 | 258 | 11.4 | Ref | - | - | - |
| Low trust | Yes | 802 | 26 | 95 | 11.8 | 1.04 | 1.02 | 0.79 | 1.33 |
| Offensive behaviour by external actors |  |  |  |  |  |  |  |  |  |
|  | No | 2542 | 83 | 291 | 11.4 | Ref | - | - | - |
| Threats and violence | Yes | 528 | 17 | 62 | 11.7 | 1.03 | 1.03 | 0.74 | 1.44 |
| Composite variables |  |  |  |  |  |  |  |  |  |
|  | No | 2239 | 73 | 264 | 11.8 | Ref | - | - | - |
| Effort-reward imbalance | Yes | 731 | 24 | 89 | 12.2 | 1.03 | 1,00 | 0,77 | 1,31 |
|  | No | 2298 | 75 | 266 | 11.6 | Ref | - | - | - |
| Job strain | Yes | 772 | 25 | 87 | 11.3 | 0.97 | 0.98 | 0.76 | 1.28 |
|  | No | 2185 | 71 | 240 | 11.0 | Ref | - | - | - |
| Low social capital | Yes | 885 | 29 | 113 | 12.8 | 1.16 | 1.15 | 0.90 | 1.47 |

All analyses were adjusted for age, sex, marital status, education, household income, occupation, workplace, seniority, part/full-time status, and number of years since last treatment.

**Supplementary Table 8.** IP-weighted mutually adjusted associations between psychosocial work factors and first-time treatment for depression (N = 21,156).

|  | Exposure status | n | % | Cases | Risk | Crude risk ratio | Mutually adjusted odds ratio | 95% CI  lower | 95% CI  upper |
| --- | --- | --- | --- | --- | --- | --- | --- | --- | --- |
| Total | - | 21156 | 100 | 350 | 1,7 | - | - | - | - |
| Interpersonal relations |  |  |  |  |  |  |  |  |  |
|  | No | 19119 | 90 | 293 | 1,5 | Ref. | - | - | - |
| Bullying | Yes | 2037 | 10 | 57 | 2,8 | 1.83 | 1.71 | 1.27 | 2.31 |
|  | No | 15894 | 75 | 251 | 1,6 | Ref. | - | - | - |
| Lack of collaboration | Yes | 5262 | 25 | 99 | 1,9 | 1.19 | 1.05 | 0.82 | 1.34 |
| Job organisation |  |  |  |  |  |  |  |  |  |
|  | No | 16053 | 76 | 256 | 1,6 | Ref. | - | - | - |
| Low control | Yes | 5103 | 24 | 94 | 1,8 | 1.16 | 1.02 | 0.78 | 1.33 |
|  | No | 18019 | 85 | 284 | 1,6 | Ref. | - | - | - |
| Low influence on schedule | Yes | 3137 | 15 | 66 | 2,1 | 1.33 | 1.23 | 0.93 | 1.63 |
|  | No | 13454 | 64 | 212 | 1,6 | Ref. | - | - | - |
| High work demands | Yes | 7702 | 36 | 138 | 1,8 | 1.14 | 1.10 | 0.88 | 1.37 |
| Management and leadership |  |  |  |  |  |  |  |  |  |
|  | No | 16122 | 76 | 254 | 1,6 | Ref. | - | - | - |
| Low justice | Yes | 5034 | 24 | 96 | 1,9 | 1.21 | 1.17 | 0.87 | 1.56 |
|  | No | 15884 | 75 | 265 | 1,7 | Ref. | - | - | - |
| Low leadership quality | Yes | 5272 | 25 | 85 | 1,6 | 0.97 | 0.98 | 0.81 | 1.36 |
|  | No | 14229 | 67 | 247 | 1,7 | Ref. | - | - | - |
| Low recognition | Yes | 6927 | 33 | 103 | 1,5 | 0.86 | 0.74 | 0.55 | 0.99 |
|  | No | 16576 | 78 | 274 | 1,7 | Ref. | - | - | - |
| Low trust | Yes | 4580 | 22 | 76 | 1,7 | 1.00 | 0.89 | 0.65 | 1.21 |
| Offensive behaviour by external actors |  |  |  |  |  |  |  |  |  |
|  | No | 18038 | 85 | 287 | 1,6 | Ref. | - | - | - |
| Threats and violence | Yes | 3118 | 15 | 63 | 2,0 | 1.27 | 1.11 | 0.81 | 1.51 |
| Composite variables^a^ |  |  |  |  |  |  |  |  |  |
|  | No | 16790 | 79 | 285 | 1,7 | Ref. | - | - | - |
| Effort-reward imbalance | Yes | 4366 | 21 | 65 | 1,5 | 0.88 | 0.86 | 0.63 | 1.15 |
|  | No | 16665 | 79 | 260 | 1,6 | Ref. | - | - | - |
| Job strain | Yes | 4491 | 21 | 90 | 2,0 | 1.28 | 1.18 | 0.90 | 1.54 |
|  | No | 16310 | 77 | 269 | 1,6 | Ref. | - | - | - |
| Low social capital | Yes | 4846 | 23 | 81 | 1,7 | 1.01 | 0.86 | 0.64 | 1.16 |

All analyses were adjusted for age, sex, marital status, education, household income, occupation seniority and part/full-time status
All analyses were weighted by the inverse probability of having missing values on at least one work-factor conditional on age, sex, marital status, education, household income, occupation, seniority, and part/full-time status.

a Analyses of these variable include the composite variables and variables not used to construct them, that is, bullying, low influence on schedule, low leadership quality and threats and violence.

**Supplementary Table 9.** IP-weighted mutually adjusted associations between psychosocial work factors and recurrent treatment for depression (N = 3070).

|  | Exposure status | n | % | Cases | Risk | Crude risk ratio | Mutually adjusted odds ratio | 95% CI  lower | 95% CI  upper |
| --- | --- | --- | --- | --- | --- | --- | --- | --- | --- |
| Total | - | 21156 | 100 | 350 | 1.7 | - | - | - | - |
| Interpersonal relations |  |  |  |  |  |  |  |  |  |
|  | No | 2623 | 85 | 284 | 10.8 | Ref. | - | - | - |
| Bullying | Yes | 447 | 15 | 69 | 15.4 | 1.43 | 1.31 | 0.96 | 1.78 |
|  | No | 2174 | 71 | 231 | 10.6 | Ref. | - | - | - |
| Lack of collaboration | Yes | 896 | 29 | 122 | 13.6 | 1.28 | 1.26 | 0.97 | 1.64 |
| Job organisation |  |  |  |  |  |  |  |  |  |
|  | No | 2161 | 70 | 230 | 10.6 | Ref. | - | - | - |
| Low control | Yes | 909 | 30 | 123 | 13.5 | 1.27 | 1.26 | 0.95 | 1.66 |
|  | No | 2543 | 83 | 282 | 11.1 | Ref. | - | - | - |
| Low influence on schedule | Yes | 527 | 17 | 71 | 13.5 | 1.21 | 1.13 | 0.84 | 1.54 |
|  | No | 1849 | 60 | 222 | 12.0 | Ref. | - | - | - |
| High work demands | Yes | 1221 | 40 | 131 | 10.7 | 0.89 | 0.85 | 0.67 | 1.09 |
| Management and leadership |  |  |  |  |  |  |  |  |  |
|  | No | 2213 | 72 | 246 | 11.1 | Ref. | - | - | - |
| Low justice | Yes | 857 | 28 | 107 | 12.5 | 1.12 | 1.15 | 0.84 | 1.58 |
|  | No | 2198 | 72 | 258 | 11.7 | Ref. | - | - | - |
| Low leadership quality | Yes | 872 | 28 | 95 | 10.9 | 0.93 | 0.69 | 0.49 | 0.96 |
|  | No | 1915 | 62 | 217 | 11.3 | Ref. | - | - | - |
| Low recognition | Yes | 1155 | 38 | 136 | 11.8 | 1.04 | 1.08 | 0.80 | 1.45 |
|  | No | 2268 | 74 | 258 | 11.4 | Ref. | - | - | - |
| Low trust | Yes | 802 | 26 | 95 | 11.8 | 1.04 | 0.93 | 0.68 | 1.28 |
| Offensive behaviour by external actors |  |  |  |  |  |  |  |  |  |
|  | No | 2542 | 83 | 291 | 11.4 | Ref. | - | - | - |
| Threats and violence | Yes | 528 | 17 | 62 | 11.7 | 1.03 | 1.01 | 0.71 | 1.42 |
| Composite variables^a^ |  |  |  |  |  |  |  |  |  |
|  | No | 2239 | 73 | 264 | 11.8 | Ref. | - | - | - |
| Effort-reward imbalance | Yes | 731 | 24 | 89 | 12.2 | 1.03 | 0.97 | 0.72 | 1.31 |
|  | No | 2298 | 75 | 266 | 11.6 | Ref. | - | - | - |
| Job strain | Yes | 772 | 25 | 87 | 11.3 | 0.97 | 0.95 | 0.71 | 1.27 |
|  | No | 2185 | 71 | 240 | 11.0 | Ref. | - | - | - |
| Low social capital | Yes | 885 | 29 | 113 | 12.8 | 1.16 | 1.24 | 0.92 | 1.68 |

All analyses were adjusted for age, sex, marital status, education, household income, occupation, workplace, seniority, part/full-time status and number of years since last treatment
All analyses were weighted by the inverse probability of having missing values on at least one work-factor conditional on age, sex, marital status, education, household income, occupation, seniority, and part/full-time status.

a Analyses of these variable include the composite variables and variables not used to construct them, that is, bullying, low influence on schedule, low leadership quality and threats and violence.

**Supplementary Table 10.** IP-weighted associations between psychosocial work factors and recurrent treatment for depression with exclusion of participants with treatment within the last year before baseline (N = 2888).

|  | Exposure status | n | % | Cases | Risk | Crude risk ratio | IPW adjusted odds ratio | 95% CI  lower | 95% CI  upper |
| --- | --- | --- | --- | --- | --- | --- | --- | --- | --- |
| Total | - | 2888 | 100 | 284 | 9.8 | - | - | - | - |
| Interpersonal relations |  |  |  |  |  |  |  |  |  |
|  | No | 2471 | 86 | 225 | 9.1 | Ref. | - | - | - |
| Bullying | Yes | 416 | 14 | 59 | 14.2 | 1.56 | 1.58 | 1.16 | 2.16 |
|  | No | 2051 | 71 | 190 | 9.3 | Ref. | - | - | - |
| Lack of collaboration | Yes | 837 | 29 | 94 | 11.2 | 1.21 | 1.25 | 0.96 | 1.63 |
| Job organisation |  |  |  |  |  |  |  |  |  |
|  | No | 2037 | 71 | 182 | 8.9 | Ref. | - | - | - |
| Low control | Yes | 851 | 29 | 102 | 12.0 | 1.34 | 1.34 | 1.03 | 1.75 |
|  | No | 2396 | 83 | 228 | 9.5 | Ref. | - | - | - |
| Low influence on schedule | Yes | 492 | 17 | 56 | 11.4 | 1.20 | 1.17 | 0.85 | 1.61 |
|  | No | 1741 | 60 | 180 | 10.3 | Ref. | - | - | - |
| High work demands | Yes | 1147 | 40 | 104 | 9.1 | 0.88 | 0.89 | 0.69 | 1.16 |
| Management and leadership |  |  |  |  |  |  |  |  |  |
|  | No | 2089 | 72 | 200 | 9.6 | Ref. | - | - | - |
| Low justice | Yes | 799 | 28 | 84 | 10.5 | 1.10 | 1.13 | 0.86 | 1.49 |
|  | No | 2078 | 72 | 211 | 10.2 | Ref. | - | - | - |
| Low leadership quality | Yes | 810 | 28 | 73 | 9.0 | 0.89 | 0.88 | 0.66 | 1.16 |
|  | No | 1808 | 63 | 175 | 9.7 | Ref. | - | - | - |
| Low recognition | Yes | 1080 | 37 | 109 | 10.1 | 1.04 | 1.07 | 0.83 | 1.37 |
|  | No | 2147 | 74 | 212 | 9.9 | Ref. | - | - | - |
| Low trust | Yes | 741 | 26 | 72 | 9.7 | 0.98 | 0.98 | 0.74 | 1.31 |
| Offensive behaviour by external actors |  |  |  |  |  |  |  |  |  |
|  | No | 2395 | 83 | 233 | 9.7 | Ref. | - | - | - |
| Threats and violence | Yes | 493 | 17 | 51 | 10.3 | 1.06 | 1.09 | 0.76 | 1.57 |
| Composite variables |  |  |  |  |  |  |  |  |  |
|  | No | 2205 | 76 | 212 | 9.6 | Ref. | - | - | - |
| Effort-reward imbalance | Yes | 683 | 24 | 72 | 10.5 | 1.10 | 1.03 | 0.77 | 1.37 |
|  | No | 2159 | 75 | 211 | 9.8 | Ref. | - | - | - |
| Job strain | Yes | 729 | 25 | 73 | 10.0 | 1.02 | 1.05 | 0.79 | 1.39 |
|  | No | 2068 | 72 | 197 | 9.5 | Ref. | - | - | - |
| Low social capital | Yes | 820 | 28 | 87 | 10.6 | 1.11 | 1.13 | 0.86 | 1.48 |

All analyses were adjusted for age, sex, marital status, education, household income, occupation seniority, part/full-time status and number of years since last treatment.
All analyses were weighted by the inverse probability of having missing values on at least one work-factor conditional on age, sex, marital status, education, household income, occupation, seniority, and part/full-time status.

**Supplementary Table 11.** IP-weighted associations between psychosocial work factors and recurrent treatment for depression with exclusion of participants with treatment within the last two years before baseline (N = 2530).

|  | Exposure status | n | % | Cases | Risk | Crude risk ratio | IPW adjusted odds ratio | 95% CI  lower | 95% CI  upper |
| --- | --- | --- | --- | --- | --- | --- | --- | --- | --- |
| Total | - | 2530 | 100 | 202 | 8.0 | - | - | - | - |
| Interpersonal relations |  |  |  |  |  |  |  |  |  |
|  | No | 2167 | 86 | 156 | 7.2 | Ref. | - | - | - |
| Bullying | Yes | 363 | 14 | 46 | 12.7 | 1.76 | 1.85 | 1.30 | 2.63 |
|  | No | 1789 | 71 | 132 | 7.4 | Ref. | - | - | - |
| Lack of collaboration | Yes | 741 | 29 | 70 | 9.4 | 1.28 | 1.33 | 0.98 | 1.80 |
| Job organisation |  |  |  |  |  |  |  |  |  |
|  | No | 1781 | 70 | 128 | 7.2 | Ref. | - | - | - |
| Low control | Yes | 749 | 30 | 74 | 9.9 | 1.37 | 1.37 | 1.02 | 1.85 |
|  | No | 2107 | 83 | 166 | 7.9 | Ref. | - | - | - |
| Low influence on schedule | Yes | 423 | 17 | 36 | 8.5 | 1.08 | 1.14 | 0.78 | 1.66 |
|  | No | 1519 | 60 | 127 | 8.4 | Ref. | - | - | - |
| High work demands | Yes | 1011 | 40 | 75 | 7.4 | 0.89 | 0.90 | 0.67 | 1.20 |
| Management and leadership |  |  |  |  |  |  |  |  |  |
|  | No | 1819 | 72 | 137 | 7.5 | Ref. | - | - | - |
| Low justice | Yes | 711 | 28 | 65 | 9.1 | 1.21 | 1.24 | 0.91 | 1.69 |
|  | No | 1804 | 71 | 147 | 8.1 | Ref. | - | - | - |
| Low leadership quality | Yes | 726 | 29 | 55 | 7.6 | 0.93 | 0.92 | 0.67 | 1.27 |
|  | No | 1575 | 62 | 120 | 7.6 | Ref. | - | - | - |
| Low recognition | Yes | 955 | 38 | 82 | 8.6 | 1.13 | 1.14 | 0.85 | 1.52 |
|  | No | 1865 | 74 | 150 | 8.0 | Ref. | - | - | - |
| Low trust | Yes | 665 | 26 | 52 | 7.8 | 0.97 | 0.93 | 0.67 | 1.30 |
| Offensive behaviour by external actors |  |  |  |  |  |  |  |  |  |
|  | No | 2102 | 83 | 163 | 7.8 | Ref. | - | - | - |
| Threats and violence | Yes | 428 | 17 | 39 | 9.1 | 1.18 | 1.15 | 0.76 | 1.73 |
| Composite variables |  |  |  |  |  |  |  |  |  |
|  | No | 1933 | 76 | 147 | 7.6 | Ref. | - | - | - |
| Effort-reward imbalance | Yes | 597 | 24 | 55 | 9.2 | 1.21 | 1.16 | 0.84 | 1.59 |
|  | No | 1886 | 75 | 148 | 7.8 | Ref. | - | - | - |
| Job strain | Yes | 644 | 25 | 54 | 8.4 | 1.07 | 1.07 | 0.78 | 1.48 |
|  | No | 1769 | 70 | 135 | 7.6 | Ref. | - | - | - |
| Low social capital | Yes | 734 | 29 | 67 | 9.1 | 1.20 | 1.23 | 0.90 | 1.67 |

All analyses were adjusted for age, sex, marital status, education, household income, occupation seniority, part/full-time status and number of years since last treatment.
All analyses were weighted by the inverse probability of having missing values on at least one work-factor conditional on age, sex, marital status, education, household income, occupation, seniority and part/full-time status
